# Modulation of u-PA, MMPs and their inhibitors by a novel nutrient mixture in human female cancer cell lines

**DOI:** 10.3892/or.2012.1879

**Published:** 2012-06-20

**Authors:** M. WAHEED ROOMI, TATIANA KALINOVSKY, MATTHIAS RATH, ALEKSANDRA NIEDZWIECKI

**Affiliations:** Dr. Rath Research Institute, Santa Clara, CA, USA

**Keywords:** breast cancer MDA-MB-231 and MCF-7, cervical cancer HeLa, MMP-2 and MMP-9, nutrient mixture, ovarian cancer SK-OV-3, tissue inhibitors of metalloproteinases, urokinase plasminogen activators, uterine cancer SK-UT-1

## Abstract

Cancers of the breast, cervix, uterus and ovary are the most prevalent cancers in women worldwide. Proteases play a key role in tumor cell invasion and metastasis by digesting the basement membrane and ECM components. Strong clinical and experimental evidence demonstrates association of elevated levels of urokinase plasminogen activators (u-PA) and matrix metalloproteinases (MMPs) with cancer progression, metastasis and shortened patient survival. MMP activities are regulated by specific tissue inhibitors of metalloproteinases (TIMPs). Our main objective was to study the effect of a nutrient mixture (NM) on the activity of u-PA, MMPs and TIMPs in human breast, cervix, uterine and ovarian cancer cell lines. Human breast (MDA-MB-231 and MCF-7), cervical (HeLa), uterine (SK-UT-1) and ovarian (SKOV3) cancer cell lines were cultured in their respective media and treated at confluence with NM at 0, 50, 100, 250, 500 and 1000 μg/ml. Analysis of u-PA activity was carried out by fibrin zymography, MMPs by gelatinase zymography and TIMPs by reverse zymography. Both breast and uterine cancer cell lines expressed u-PA, which was inhibited by NM in a dose-dependent manner. However, no bands corresponding to u-PA were detected for HeLa and SK-OV-3 cell lines. On gelatinase zymography, MDA-MB-231 and MCF-7 showed one band corresponding to MMP-9, HeLa showed two bands, an intense band corresponding to MMP-2 and a faint band corresponding to MMP-9, SK-UT-1 showed PMA-induced MMP-9, and SK-OV-3 showed a band corresponding to MMP-2. NM inhibited their expression in all cell lines. The activity of TIMPs was upregulated in all cancer cell lines in a dose-dependent manner. Analysis revealed a positive correlation between u-PA and MMPs and a negative correlation between u-PA/MMPs and TIMPs. These findings suggest the therapeutic potential of NM in the treatment of female cancers.

## Introduction

Breast cancer is the most prevalent cancer in women worldwide, and the leading cause of cancer death in women, accounting for 23% of the total new cancer cases and 14% of total cancer deaths (1). About 12% of women in the United States will develop invasive breast cancer over the course of their lifetime. About 39,520 women in the US were expected to die in 2011 from breast cancer (2). Though treatable in early stages, once metastasis has occurred the survival rate is drastically reduced to a median of 2–3 years and treatment focuses on palliative care (3). Endometrial cancer is the most common cancer of the female reproductive organs, with most cases diagnosed in the 50 to 69 age group. Approximately 46, 470 new cases are estimated to be diagnosed in 2011 and 8,120 deaths (4). Cervical cancer is the third most commonly diagnosed cancer and the fourth leading cause of deaths in females worldwide, with more than 85% of associated deaths occurring in developing countries (1). Cervical cancer develops slowly, taking 10–15 years to develop into cancer from a pre-cancerous condition called dysplasia. Though fully treatable in early stages, once the cancer has metastasized, patient outcome is poor. Ovarian carcinoma, which occurs mainly in post-menopausal women, is the ninth most common cancer among women and the fifth leading cause of death among women worldwide (5). Since ovarian cancer often remains clinically silent, the majority of patients with ovarian carcinoma have advanced intraperitoneal metastatic disease at diagnosis, resulting in a poor prognosis. About 80% of ovarian cancer cases are diagnosed at an advanced stage after metastasis has occurred (5).

Progression of metastasis occurs secondary to cancer cell detachment from the primary tumor, basement membrane degradation, cancer cell invasion into the surrounding stroma, and entry into and transport through the vascular or lymphatic system to distal sites such as the liver, lungs, and brain, and extravasation, tumor cell proliferation and angiogenesis at distal sites (6–10). Tumor cell invasion depends upon degradation of the extracellular matrix (ECM), which is composed of collagen, proteoglycans, fibronectin, laminin and other glycoproteins, and, when intact, acts as a barrier to block cancer cell invasion (11–13). Two families of proteases, the MMPs and urokinase plasminogen activators (u-PA) are involved in tumor invasion and metastasis. Numerous clinical and experimental studies have demonstrated that elevated levels of u-PA and MMPs are associated with tumor growth, cancer progression, metastasis and shortened survival in patients (14–27).

MMPs, especially MMP-2 and MMP-9 play key roles in tumor cell invasion and metastasis due to their ability to degrade type IV collagen, a major component of the ECM (13,28,29). Secreted in their latent zymogenic form as inactive pro-enzymes, MMP-2 and MMP-9 are cleaved by other MMPs or proteases to yield the activated forms of 68, 58, and 54 kDa for MMP-2, and 84 kDa for MMP-9. Proteolytic activities of MMP-2 and MMP-9 are inhibited by specific inhibitors, tissue inhibitors of metalloproteinases (TIMPs). Thus, a critical determinant of net proteolytic degradation is the balance between MMP and TIMP levels. Clinical studies note the association of MMP expression with progression of breast (18–20), cervical (21,22), uterine (24) and ovarian (23,25) cancers.

The serine protease u-PA consists of two disulfide bridges linked to polypeptides, which are cleaved to the active chain (33kDa) by various stimuli. The protease u-PA converts plasminogen to plasmin, which is capable of promoting tumor growth and angiogenesis, degrading the ECM and basement membrane and activating pro-MMPs (30). Synthetic u-PA inhibitors have been reported to inhibit metastasis of mammary carcinoma cell lines (31). Clinical studies have shown that high uPA levels are correlated with progression of female cancers (25–27,32,33).

Rath and Pauling (34) proposed that nutrients such as lysine and ascorbic acid be utilized to target plasmin-mediated connective tissue degradation as a universal approach to tumor growth and expansion. Binding to plasminogen active sites, lysine blocks plasminogen activation into plasmin by tissue plasminogen activator (t-PA). Thus it modulates the plasmin-induced MMP activation cascade (35). Subsequent studies confirmed this approach and resulted in identifying a novel formulation composed of lysine, ascorbic acid, proline and green tea extract and other micronutrients (NM), which has shown significant anticancer activity against a large number (~40) of cancer cell lines, blocking cancer growth, tissue invasion and MMP expression both *in vitro* and *in vivo*(36). In this study, we focused on the modulating effect of NM on the activities of MMP-2 and -9, TIMPs and u-PA in human breast (MBA-MD-231, MCF-7), cervical (HeLa), uterine (SK-UT-1) and ovarian (SK-OV-3) cancer cell lines.

## Methods and materials

### Materials

Human breast cancer cells MDA-MB-231 and MCF-7, cervical cancer cells HeLa, uterine leimyosarcoma SK-UT-1 and ovarian cancer cells SK-OV-3, along with their culture media were obtained from ATCC. Antibiotics, penicillin, and fetal bovine serum (FBS), were obtained from Gibco (BRL, Long Island, NY). Twenty-four-well tissue culture plates were obtained from Costar (Cambrdige, MA). Gelatinase zymography was performed in 10% Novex pre-cast SDS polyacrylamide gel (Invitrogen Inc.) with 0.1% gelatin in non-reducing conditions. The nutrient mixture (NM), prepared by VitaTech (Hayward, CA) was composed of the following ingredients in the relative amounts indicated: vitamin C (as ascorbic acid and as Mg, Ca, and palmitate ascorbate) 700 mg; L-lysine 1000 mg; L-proline 750 mg; L-arginine 500 mg; N-acetyl cysteine 200 mg; standardized green tea extract (80% polyphenol) 1000 mg; selenium 30 μg; copper 2 mg; manganese 1 mg. All other reagents used were of high quality and were obtained from Sigma, unless otherwise indicated.

### Cell cultures

Human breast cancer cell lines MDA-MB-231 and MCF-7 and cervical cancer cell line HeLa were grown in MEM, uterine cell line SK-UT-1 in DEME and ovarian cancer cell line SK-OV-3 in McCoy medium supplemented with 10% FBS, penicillin (100 U/ml), and streptomycin (100 μg/ml) in 24-well tissue culture plates. The cells were plated at a density of 1×10^5^ cells/ml and grown to confluency in a humidified atmosphere at 5% CO_2_ at 37°C. Serum-supplemented media were removed and the cell monolayer was washed once with PBS with the recommended serum-free media. The cells were treated with the nutrient mixture, dissolved in media and tested at 0, 50, 100, 250, 500, and 1000 μg/ml in triplicate at each dose. Parallel sets of cultures were treated with PMA (100 ng/ml) for induction of MMP-9. Control and PMA treatments were done in triplicates. The plates were then returned to the incubator. The conditioned media were collected separately, pooled, and centrifuged at 4°C for 10 min at 3000 rpm to remove cells and cell debris. The supernatant was collected and used to assess for u-PA activity (by fibrin zymography on 10% SDS-PAGE gels containing fibrinogen and plasminogen), MMP-2 and -9 (by gelatinase zymography), and TIMPs (by reverse zymography).

### Fibrin zymography

Fibrin zymography was used to analyze u-PA activity on 10% SDS-PAGE gels containing fibrinogen (5.5 mg/ml) and plasminogen (50 μg/ml). After electrophoresis, the gels were washed twice with 2.5% Triton X-100 for 30 min. The gels were then incubated overnight at 37°C with 0.1% glycine buffer pH 7.5 and then stained with 0.5% Coomassie Brilliant Blue R250 and destained. Electrophoresis of u-PA and t-PA were conducted for comparison. Fibrin zymograms were scanned using CanoScan 9950F Canon Scanner.

### Gelatinase zymography

Gelatinase zymography was performed in 10% NOVEX Pre-Cast SDS polyacrylamide gel (Invitrogen Corporation) in the presence of 0.1% gelatin under non-reducing conditions. Culture media (20 μl) were mixed with sample buffer and loaded for SDS-PAGE with tris glycine SDS buffer as suggested by the manufacturer (Novex). Samples were not boiled before electrophoresis. Following electrophoresis the gels were washed twice in 2.5% Triton X-100 for 30 min at room temperature to remove SDS. The gels were then incubated at 37°C overnight in substrate buffer containing 50 mM Tris-HCl and 10 mM CaCl_2_ at pH 8.0 and stained with 0.5% Coomassie Blue R250 in 50% methanol and 10% glacial acetic acid for 30 min and destained. Upon renaturation of the enzyme, the gelatinases digest the gelatin in the gel and give clear bands against an intensely stained background. Protein standards were run concurrently and approximate molecular weights were determined by plotting the relative mobilities of known proteins.

### Reverse zymography

TIMPS were analyzed by reverse zymography on 15% SDS gels containing serum-free conditioned medium from cells. After electrophoresis the gels were washed twice with 2.5% Triton-X for 30 min at room temperature to remove SDS. The gels were then incubated at 37°C overnight in 50 mM Tris-HCl and 10 mM CaCl_2_ at pH 7.6 and stained with 0.5% Coomassie Blue R25, destained and scanned.

### Scanning of gelatinase and fibrin zymograms

Gelatinase and fibrin zymograms were scanned using CanoScan 9950F Canon scanner at 300 dpi. The intensity of the bands was evaluated using the pixel-based densitometer program Un-Scan-It, Version 5.1, 32-bit, by Silk Scientific Corporation (Orem, UT, USA), at a resolution of 1 Scanner Unit (1/100 of an inch for an image that was scanned at 100 dpi). The pixel densitometer calculates the optical density of each pixel (values 0 to 255) using the darkly stained background of the gel as a pixel value of 0. A logarithmic optical density scale was used since the optical density of films and gels is logarithmically proportional to the concentration. The pixel densitometer sums the optical density of each pixel to give a band’s density. In all graphs, band densities were reported as percentages of the sums of all pixels in a given lane (treatment) of a gel.

### Statistical analysis

Pearson’s correlation coefficient was determined between mean MMP-9, u-PA and TIMP-2 expression of breast cancer cell lines MDA-MB-231 and MCF-7 and uterine cell line SK-UT-1 and between mean MMP-2 and TIMP-2 expression of cervical cell line HeLa and ovarian cancer SK-OV-3 using MedCalc Software (Mariakerke, Belgium).

## Results

### Effect of NM on u-PA activity in human breast, cervical, uterine and ovarian cell lines

**Figure 1:**
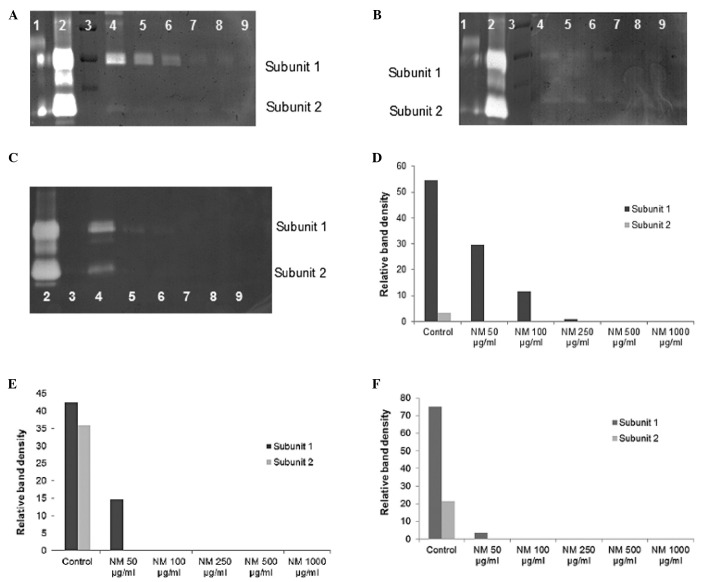
Effect of NM on breast cancer cell lines MDA-MB-231 and MCF-7 and uterine cancer cell line SK-UT-1 u-PA expression. Fibrin zymograms of MDA-MB-231 (A), MCF-7 (B) and SK-UT-1 (C) u-PA expression. Lane: 1, t-PA; 2, u-PA; 3, Markers; 4, Control, 5–9 NM 50, 100, 250, 500, 1000 μg/ml. Densitometric analyses of MDA-MB-231 (D), MCF-7 (E) and SK-UT-1 (F) u-PA expression.

Activity of u-PA was detected in both breast cancer cell lines and in the uterine cell line showing two bands corresponding to 55 and 33 kD. NM exerted dose response inhibition with virtual block of u-PA activity at 100 μg/ml in SK-UT-1 cells (linear trend R^2^=0.461) and in MCF-7 cells (linear trend R^2^=0.656), and at 500 μg/ml (linear trend R^2^=0.813) in MDA-MB-231 cells. See Fig. 1 for respective fibrin zymograms and densitometry analyses. However, no bands corresponding to u-PA were detected for HeLa and SK-OV-3 cell lines.

### Effect of NM on MMP-2 and MMP-9 expression by human breast cervical and ovarian cancer cell lines

**Figure 2:**
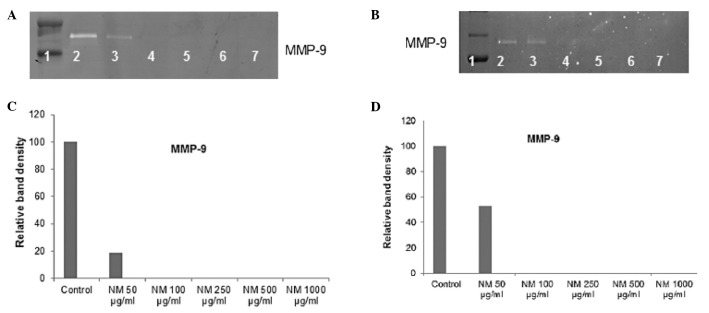
Effects of NM on breast cancer cell lines MDA-MB-231 and MCF-7 MMP-9 expression. Gelatinase zymograms of PMA-treated MDA-MB-231 cell MMP-9 secretion (A) and normal MCF-7 cell MMP-9 secretion (B). Lane: 1, Markers; 2, Control, 3–7 NM 50, 100, 250, 500, 1000 μg/ml. Densitometric analyses of PMA-treated MDA-MB-231 MMP-9 secretion (C) and normal MCF-7 MMP-9 secretion (D).

**Figure 3:**
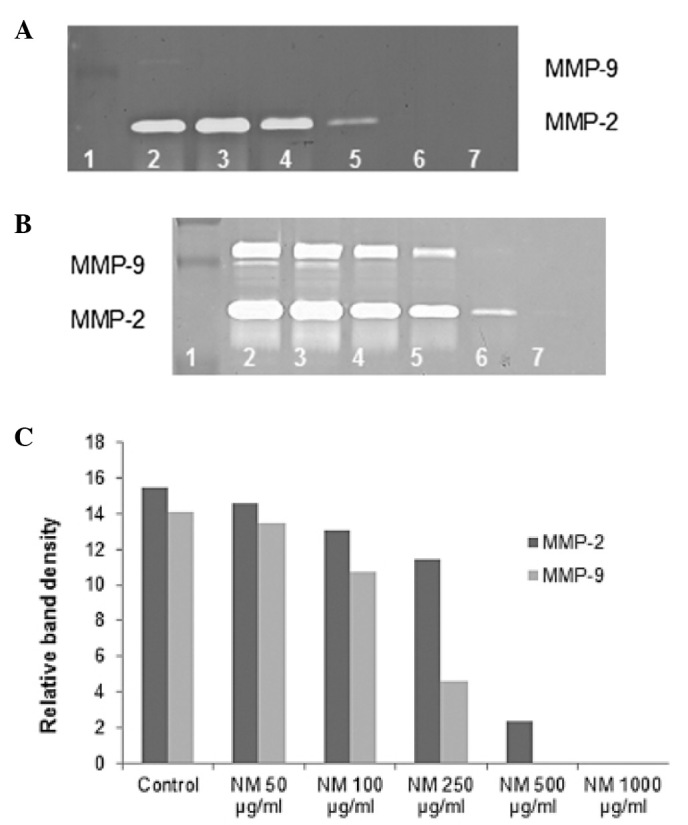
Effects of NM on cervical cancer cell line HeLa MMP-2 and MMP-9 expression. Gelatinase zymograms of normal HeLa cell MMP-2 secretion (A) and PMA-treated HeLa MMP-2 and MMP-9 secretion (B). Lane: 1, Markers; 2, Control, 3–7 NM 50, 100, 250, 500, 1000 μg/ml. Densitometric analysis of PMA-treated HeLa MMP-2 and -9 secretion (C).

**Figure 4:**
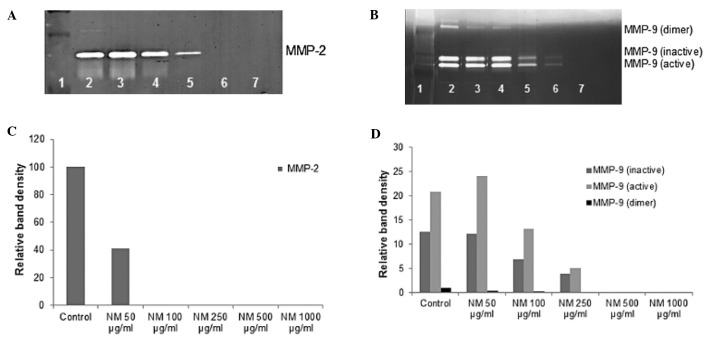
Effects of NM on ovarian cancer cell line SK-OV-3 and uterine cell line SK-UT-1 MMP-2 and -9 expression. Gelatinase zymograms of normal SK-OV-3 MMP-2 secretion (A) and PMA-treated SK-UT-1 cell MMP-9 secretion (B). Lane: 1, Markers; 2, Control, 3–7 NM 50, 100, 250, 500, 1000 μg/ml. Densitometric analyses of normal SK-OV-3 MMP-2 secretion (C) and PMA-treated SK-UT-1 MMP-9 secretion (D).

On gelatinase zymography, a band corresponding to MMP-9 was detected in both MDA-MB-231 and MCF-7 cell lines. Cervical HeLa cells showed two bands, an intense band corresponding to MMP-2 and a faint band corresponding to MMP-9, which was enhanced with PMA treatment. Normal uterine SK-UT-1 cells did not express MMP-2 or MMP-9; however, MMP-9 was induced with PMA. Ovarian SK-OV-3 cells showed only a band corresponding to MMP-2. NM inhibited MMP expression in all cell lines, with complete block of MMP-9 in breast cancer cells at 100 μg/ml and in uterine and cervical cells at 500 μg/ml. NM blocked MMP-2 expression in ovarian and cervical cell lines at 100 and 1000 μg/ml, respectively. See Figs. 2–4 for gelatinase zymograms and densitometry analyses.

### Effect of NM on TIMPs activity in human breast, cervical and ovarian cancer cell lines

**Figure 5:**
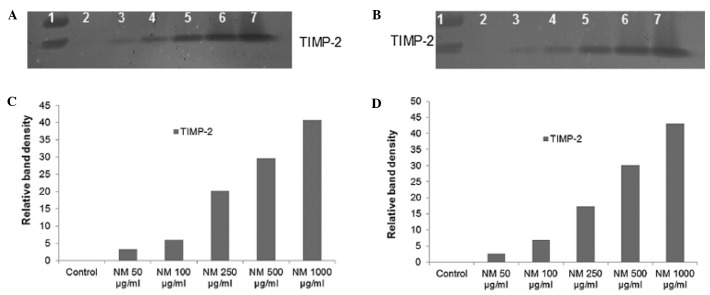
Effect of NM on breast cancer cell lines MDA-MB-231 and MCF-7 TIMP-2 expression. Gelatinase zymograms of MDA-MB-231 TIMP-2 expression (A) and MCF-7 TIMP-2 expression (B). Lane: 1, Markers; 2, Control, 3–7 NM 50, 100, 250, 500, 1000 μg/ml. Densitometric analyses of MDA-MB-231 TIMP-2 expression (C) and MCF-7 TIMP-2 expression (D).

**Figure 6:**
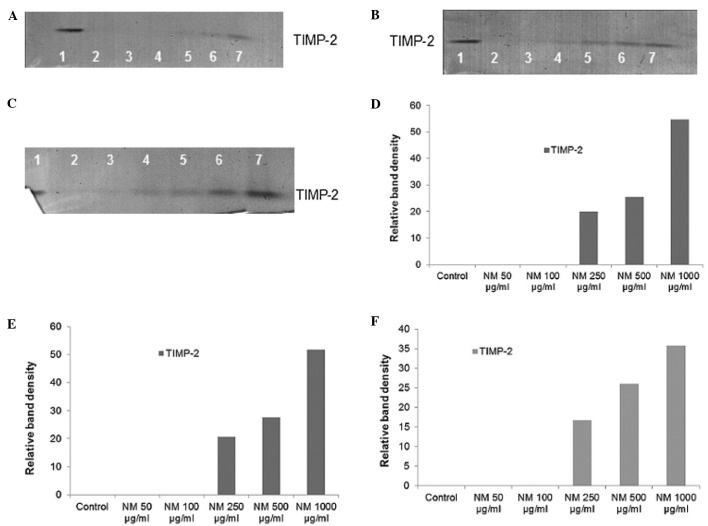
Effect of NM on cervical cancer cell line HeLa, ovarian cancer cell line SK-OV-3 and uterine cancer cell line SK-UT-1 TIMP-2 expression. Gelatinase zymograms of HeLa TIMP-2 expression (A) SK-OV-3 TIMP-2 expression (B) and SK-UT-1 TIMP-2 expression (C) Lane: 1, Markers; 2, Control, 3–7 NM 50, 100, 250, 500, 1000 μg/ml. Densitometric analyses of HeLa TIMP-2 expression (D), SK-OV-3 TIMP-2 expression (E) and SK-UT-1 TIMP-2 expression (F).

Reverse zymography revealed up regulation of TIMP-2 activity with NM treatment in all cancer cell lines in a dose-dependent manner. Minimum activity was expressed at 50 and maximum at 1000 μg/ml NM. See Figs. 5 and 6 for respective reverse zymograms and densitometry analyses.

### Correlation between female cancer cell lines u-PA, TIMP-2 and MMP expressions

**Figure 7:**
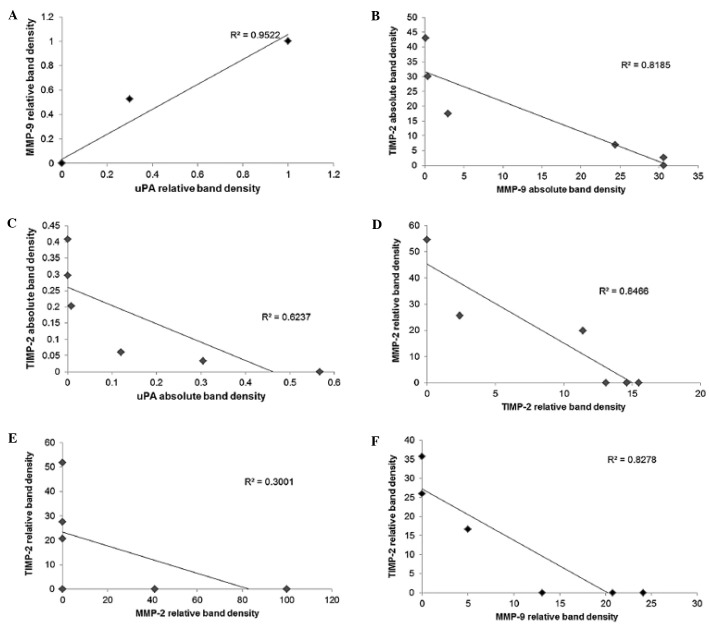
(A) Correlation between the effects of NM on breast cancer MCF-7 u-PA and MMP-9 expression. (B) Correlation between the effects of NM on breast cancer MCF-7 TIMP-2 and MMP-9 expression. (C) Correlation between the effects of NM on breast cancer MDA-MB-231 TIMP-2 and u-PA expression. (D) Correlation between the effects of NM on cervical cancer HeLa MMP-2 and TIMP-2 expression. (E) Correlation between the effects of NM on ovarian cancer SK-OV-3 TIMP-2 and MMP-2 expression. (F) Correlation between the effects of NM on uterine cancer SK-UT-1 MMP-9 and u-PA expression.

Analysis revealed a positive correlation between NM-treated breast cancer cell line MCF-7 u-PA and MMP expressions, as shown in Fig. 7A, with a correlation coefficient r=0.976. A negative correlation (correlation coefficient r= −0.904) was found between the expressions of MCF-7 u-PA and MMP-9 (Fig. 7B). A negative correlation (correlation coefficient r= −0.790) was found between MBA-MB-231 expression of TIMP-2 and u-PA (Fig. 7C). Negative correlations were found between HeLa expression of TIMP-2 and MMP-2 (correlation coefficient r= −0.820) and between SK-OV-3 TIMP-2 and MMP-2 (correlation coefficient r= −0.548), as shown in Fig. 7D and E, respectively. A negative correlation was found between uterine SK-UT-1 cell TIMP-2 and MMP-9 (r= −0.910), as shown in Fig. 7F; Table I).

## Discussion

Critical events in tumor cell invasion include cell attachment, degradation of the ECM and migration through the disrupted matrix. The two families of proteases, matrix metalloproteinases and urokinase plasminogen activators play key roles in tumor cell invasion. Experimental studies have demonstrated the role of urokinase plasminogen, especially cell surface u-PA, as an initiator of ECM proteolysis and associated tumor cell invasion (35). The protease u-PA converts plasminogen to plasmin, which is capable of promoting tumor growth and angiogenesis, degrading the ECM and basement membrane and activating pro-MMPs (30). Duffy first reported the prognostic value of u-PA in breast cancer patients, showing a positive correlation between high levels of u-PA and cancer progression (32). High u-PA levels have been reported to be prognostic indicators of increased risk of endometrial cancer progression (26,27). High levels of u-PA in cervical cancer patients have also been reported to be correlated with pelvic lymph node metastasis (17) and were reported to predict survival in advanced ovarian cancer patients after radical surgery and chemotherapy (33). Matrix metalloproteinases, especially MMP-2 and MMP-9 play pivotal roles in tumor cell invasion and metastasis due to their ability to degrade type IV collagen, a major component of the ECM. Overproduction of MMPs, especially MMP-2 and -9 has been associated with a more aggressive behavior of female cancers (18,22–24).

Our study demonstrated that the specific mixture of nutrients tested significantly inhibited breast cancer cell MDA-MB-231 and MCF-7 u-PA secretion. (Cervical cancer HeLa and ovarian cancer SK-OV-3 cells were not found to secrete u-PA in this study). Furthermore, the NM demonstrated dose-dependent decrease in MMP secretion and increase in TIMP-2 secretion by all these female cancer cells. As expected, a significant positive correlation was found between the secretion of u-PA and MMPs and a significant negative correlation between u-PA and TIMP-2 secretion by NM treatment of breast cancer cells. As anticipated, a significant negative correlation was found between MMP and TIMP-2 secretion by all the female cancer cell lines tested. Furthermore, a previous study demonstrated significant correlation between NM inhibition of Matrigel invasion and NM modulation of the MMP-2 and -9 activity of the female cancer cells lines studied (37). A significant negative correlation was found between NM modulation of Matrigel invasion inhibition and MMP-9 secretion with breast cancer MDA-MB-231 (r= − 0.851) and MCF-7 (r= −0.993) cell lines and with uterine cancer SK-UT-1 cell line (r= −0.910). For cervical HeLa cells and ovarian SK-OV-3 cells, negative correlations (r= −0.924 and r= −0.812, respectively) were found between NM modulation of Matrigel invasion inhibition and MMP-2 secretion. A previous *in vivo* study of the effects of NM on breast cancer supports these results in that it demonstrated significant inhibition of MDA-MB-231 xenograft tumor growth in nude mice and inhibition of MMP-9 and VEGF secretion and mitosis in the tissue of nutrient-supplemented mice (38).

In contrast to the associated toxicity and limited efficacy of standard cancer chemotherapy and radiation therapy, extensive research has documented the efficacy and safety of dietary and botanical natural compounds in cancer prevention (39). The nutrient mixture was formulated by selecting nutrients that act on critical physiological targets in cancer progression and metastasis, as documented in both clinical and experimental studies. Combining these micronutrients expands metabolic targets, maximizing biological impact with lower doses of components. For example, a previous study of the comparative effects of NM, green tea extract and EGCG on inhibition of MMP-2 and MMP-9 secretion of different cancer cell lines with varying MMP secretion patterns, documented the superior potency of NM over GTE and EGCG at equivalent doses (40). These results can be understood from the more comprehensive treatment offered by the combination of nutrients in NM over individual components of NM since MMP-2 and MMP-9 are mediated by differential pathways.

Optimal ECM structure depends upon adequate supplies of ascorbic acid and the amino acids lysine and proline to ensure proper synthesis and hydroxylation of collagen fibers. In addition, lysine contributes to ECM stability as a natural inhibitor of plasmin-induced proteolysis (34,41). Manganese and copper are also essential for collagen formation. There is considerable documentation of the potency of green tea extract in modulating cancer cell growth, metastasis, angiogenesis, and other aspects of cancer progression (42–48). N-acetyl cysteine and selenium have demonstrated inhibition of tumor cell MMP-9 and invasive activities, as well as migration of endothelial cells through ECM (49–51). Ascorbic acid demonstrates cytotoxic and antimetastatic actions on malignant cell lines (52–56) and cancer patients have been found to have low levels of ascorbic acid (57,58). Low levels of arginine, a precursor of nitric oxide (NO), can limit the production of NO, which has been shown to predominantly act as an inducer of apoptosis (59).

In conclusion, the NM demonstrated potent anticancer activity by targeting primary mechanisms responsible for the aggressive spread of breast, uterine, cervical and ovarian cancer. In this *in vitro* study, the NM significantly inhibited breast cancer cell lines MDA-MB-231 and MCF-7 and uterine cell line SK-UT-1 secretion of u-PA and MMP-9 and increased their secretion of TIMP-2, suggesting its potential in modulating breast and uterine cancer invasion and metastasis. Cervical HeLa and ovarian SK-OV-3 cell lines did not secrete u-PA; however, secretion by these cell lines of MMP-2 was inhibited by NM and secretion of TIMP-2 was enhanced by NM. With all these female cancer cell lines, NM inhibition of MMP secretion was found to be correlated significantly with Matrigel invasion of these cell lines. Furthermore, use of the nutrient mixture would not pose any toxic effect clinically, especially in the relevant doses, as *in vivo* safety studies demonstrate. An *in vivo* toxicology study showed that NM had no adverse effects on vital organs (heart, liver, and kidney), or on the associated functional serum enzymes (60).

## Figures and Tables

**Table I:** Overview of MMP-2 and -9, u-PA and TIMP-2 expression of female cancer cell lines.

Cancer cell line	MMP-2	MMP-9	u-PA	TIMP-2
Breast cancer MDA-MB-231	−	+	+	+
Breast cancer MCF-7	−	+	+	+
Cervical cancer HeLa	+	+	−	+
Ovarian cancer SK-OV-3	+	−	−	+
Uterine cancer SK-UT-1	−	With PMA induction	+	+
